# Quantum-parallel vectorized data encodings and computations on trapped-ion and transmon QPUs

**DOI:** 10.1038/s41598-024-53720-x

**Published:** 2024-02-10

**Authors:** Jan Balewski, Mercy G. Amankwah, Roel Van Beeumen, E. Wes Bethel, Talita Perciano, Daan Camps

**Affiliations:** 1 National Energy Research Scientific Computing Center, Lawrence Berkeley National Laboratory, Berkeley, CA 94720 USA; 2 Department of Mathematics, Applied Mathematics and Statistics, Case Western Reserve University, Cleveland, OH 44106 USA; 3 Applied Mathematics and Computational Research Division, Lawrence Berkeley National Laboratory, Berkeley, CA 94720 USA; 4 Computer Science Department, San Francisco State University, San Francisco, CA 94132 USA; 5 Scientific Data Division, Lawrence Berkeley National Laboratory, Berkeley, CA 94720 USA

**Keywords:** Quantum information, Applied mathematics, Computer science, Scientific data, Genomics

## Abstract

Compact data representations in quantum systems are crucial for the development of quantum algorithms for data analysis. In this study, we present two innovative data encoding techniques, known as **QCrank** and **QBArt**, which exhibit significant quantum parallelism via uniformly controlled rotation gates. The **QCrank** method encodes a series of real-valued data as rotations on data qubits, resulting in increased storage capacity. On the other hand, **QBArt** directly incorporates a binary representation of the data within the computational basis, requiring fewer quantum measurements and enabling well-established arithmetic operations on binary data. We showcase various applications of the proposed encoding methods for various data types. Notably, we demonstrate quantum algorithms for tasks such as DNA pattern matching, Hamming weight computation, complex value conjugation, and the retrieval of a binary image with 384 pixels, all executed on the Quantinuum trapped-ion QPU. Furthermore, we employ several cloud-accessible QPUs, including those from IBMQ and IonQ, to conduct supplementary benchmarking experiments.

## Introduction

Quantum computing is believed to open doorways to novel methods and algorithms that can outperform their classical counterparts^1^. Among the most prominent examples of quantum algorithms are Shor’s prime factoring algorithm^2^ and Grover’s unstructured search algorithm^3^. In addition, recent results show that quantum computers have great potential to solve problems in machine learning^4–9^. Similarly, there has been considerable work in quantum image processing^10–14^. However, despite this progress, the current era of noisy intermediate-scale quantum (NISQ) devices still calls for basic research to understand better the capabilities and applicability of quantum information science (QIS)^15^.

A crucial problem when designing and implementing quantum algorithms that process classical data is the *data encoding problem*^16^, which relates to how data is encoded in the quantum state of a qubit register, and is closely related to the data input problem. In the encoding process, there is a trade-off between the efficient use of the Hilbert space and the computational complexity of the algorithms leveraging the quantum representation^9^.

The main contributions of this paper are related to the data encoding problem and how to address it on NISQ hardware to implement quantum data analysis algorithms in practice. First, we introduce an extension of the uniformly controlled rotation gate^17^ that enables concurrent execution of two-qubit CX gates on the address and data qubits of the memory. We call this a parallel uniformly controlled rotation gate and it can be viewed as a generalization of the parallel Toffoli gate decomposition^18^ to more than 2 address lines.

We implement our circuits in two quantum data encoding schemes: a **QCrank** angle encoding for continuous data and a **QBArt** basis encoding for discrete data in binary representation. Second, we present the results of a collection of data encoding and analysis experiments demonstrated using real quantum processors at an unprecedented scale for different data types, including images, DNA sequences, and time-series.

The three most well-known types of data encoding are basis encoding, amplitude encoding, and angle encoding^19^. Assume that the input data is an $N = 2^n$-dimensional vector $\vec {x}= \left[ x_0, \ldots , x_{N-1} \right]$. *Basis encoding* is mainly used when discrete data must be arithmetically manipulated in a quantum algorithm. In this case, $\vec {x}$ is a binary string obtained from the original classical data. For example, if the classical data is the vector [0, 1, 2, 3], then $\vec {x}= [00,01,10,11]$. This binary string is encoded in the computational basis states of a qubit system, i.e., $|\vec {x}\rangle = |00011011 \rangle$. In the case of *amplitude encoding*, a (normalized) real- or complex-valued data vector $\vec {x}$ is directly encoded in a $2^n$-dimensional Hilbert space through the amplitudes of the state $\sum _i x_i \left| {i}\right\rangle$. Finally, in an *angle encoding*, each $x_i$ in $\vec {x}$ is embedded through single-qubit rotations, for example, as $\bigotimes _i \left( \cos (x_i/2) \left| {0}\right\rangle + \sin (x_i/2) \left| {1}\right\rangle \right)$ in case of a Pauli-*Y* rotation.

As previously mentioned, the problem of quantum data encoding is closely related to research on quantum data input and quantum memory. Analogous to classical computer memory, the two main designs under investigation within the field of quantum computing are quantum read only memory (QROM)^20^ and quantum random access memory (QRAM)^18,21^. Both are based on coherent access to data stored in quantum memory addresses that can be queried within a quantum algorithm. Theoretical designs vary from quantum circuit implementations to proposals for native hardware implementations of quantum memory.

This work extends data encodings predominantly used in quantum image processing, usually called quantum image representations (QIR), and have a close connection to QROM/QRAM. A variety of QIR methods have been developed^22^. The (improved) flexible representation of quantum images ([I]FRQI)^23–25^, the (improved) novel enhanced quantum representation ([I]NEQR)^26,27^, the multi-channel representation of quantum images (MCRQI/MCQI)^28,29^, and the (improved) novel quantum representation of color digital images ([I]NCQI)^30,31^ are among the most powerful existing QIR methods. Our previous work proposed an overarching encoding framework called QPIXL^32^ that unifies all QIRs mentioned above. In the QPIXL framework, every QIR can be written as

1 $$\begin{aligned} \left| {\psi (\vec {x})}\right\rangle = \sum _i \left| {i}\right\rangle \otimes \left| {c_i}\right\rangle , \end{aligned}$$

where $\left| {c_i}\right\rangle$ is an encoding of the pixel colors in the qubit state and $\left| {i}\right\rangle$ an encoding of the pixel positions in the qubit state^32^. All QIRs that are commonly considered in the literature use a straightforward basis encoding for the pixel position information. However, the color mapping varies for different QIRs. For example, NEQR employs a basis encoding for the pixel color information, FRQI uses an angle encoding in a single qubit. In contrast, IFRQI and MCRQI/MCQI use angle encodings over multiple qubits.

The second contribution of QPIXL is an asymptotically optimal quantum circuit implementation to prepare QIRs based on *uniformly controlled rotation* ($\text {UCR}$) gates^17^. A $\text {UCR}$ is a multi-parameter, multi-qubit gate acting on $n_a$ control or *address qubits* and 1 target or *data qubit*. A UCR gate performs a single-qubit rotation of the data qubit around a fixed axis on the Bloch sphere. Here, the rotation angle depends conditionally on the computational basis state of the address qubits. As such, it is parametrized by $2^{n_a}$ rotation angles as there are $2^{n_a}$ different basis states in the address register. For example, assuming Pauli-*Y* rotations,

2 $$\begin{aligned} R_y(\phi ):= e^{-\text {i} Y \phi /2} = \begin{bmatrix} \cos \frac {\phi }{2} &{} -\sin \frac {\phi }{2} \\ \sin \frac {\phi }{2} &{} \cos \frac {\phi }{2} \\ \end{bmatrix}, \end{aligned}$$

the unitary matrix corresponding to a $\text {UCR}_{y}$ gate with the final qubit as the data qubit is given by the following block diagonal matrix,

3 $$\begin{aligned} \small { \text {UCR}_{y}(\vec {\alpha }) = \begin{bmatrix} R_y(\alpha _0) &{} &{} \\ &{} \ddots &{} \\ &{} &{} R_y(\alpha _{2^{n_a}-1}) \end{bmatrix}, } \end{aligned}$$

where $\vec {\alpha }= \left[ \alpha _0, \ldots , \alpha _{2^{n_a}-1} \right]$ is a vector of rotation angles. It follows that^32^

4 $$\begin{aligned} \left| {\psi _{\text {FRQI}}(\vec {\alpha })}\right\rangle&= \text {UCR}_{y}(\vec {\alpha }) \, (H^{\otimes n_a} \otimes I) \, \left| {0}\right\rangle ^{\otimes (n_a + 1)}, \nonumber \\&= \sum _i \left| {i}\right\rangle \otimes (\cos \frac {\alpha _i}{2} \left| {0}\right\rangle + \sin \frac {\alpha _i}{2} \left| {1}\right\rangle ). \end{aligned}$$

To recover $\vec {\alpha }$ through projective measurement of $\left| {\psi _{\text {FRQI}}(\vec {\alpha })}\right\rangle$ in the computational basis, we sample from the probability density function (PDF) $\big |\!\left| {\psi _{\text {FRQI}}(\vec {\alpha })}\right\rangle \!\big |^2 = \big |\!\left[ c_{0}, s_{0}, \ldots , c_{2^{n_a}-1}, s_{2^{n_a}-1} \right] \!\big |^2$, where $c_i = \cos \frac {\alpha _i}{2}$ and $s_i = \sin \frac {\alpha _i}{2}$. The input angles $\vec {\alpha }$ can be uniquely recovered by measuring the PDF,

5 $$\begin{aligned} \alpha ^{\text {meas}}_{i}=2 \arctan {\sqrt{\frac{|s_{i}|^2}{|c_{i}|^2}}}, \qquad i \in [2^{n_a}], \end{aligned}$$

provided that $\alpha _i \in [0, \pi ]$. The input data $\vec {x}$ should be rescaled to rotation angles in this restricted range, e.g., $\alpha _i = x_i / A$, where *A* is a scaling factor such that the angles are mapped to $[0, \pi ]$.

**Figure 1:**
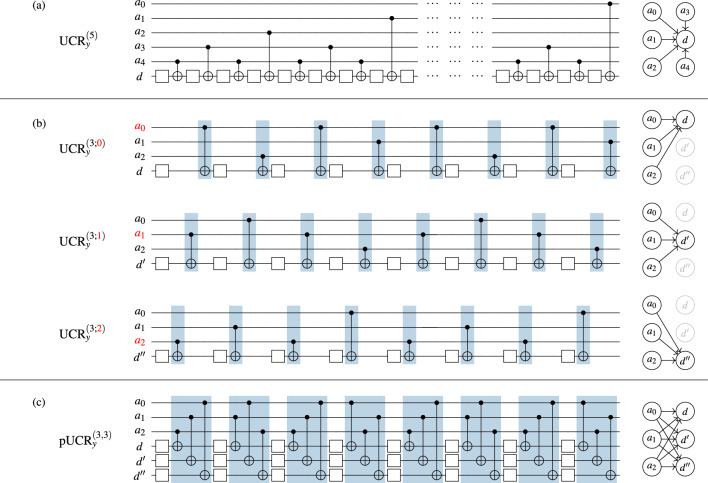
Different types of uniformly controlled rotation (UCR) gates with optimal connectivity graphs for qubits shown on the right. (**a**) Standard compact circuit implementation for a $\text {UCR}_{y}$ gate that was used in the QPIXL framework^32^ for 5 address and 1 data qubits. Square boxes denote single qubit $R_y$ rotations. (**b**) All 3 possible realizations of the cyclic permuted UCRs for 3 address and 1 data qubits. (**c**) Parallel UCR for 3 address and 3 data qubits. The same 3 different permuted $\text {UCR}_{y}$ circuits using the common 3 address qubits and 3 different data qubits can be reordered to an equivalent circuit with the same CX depth as a single $\text {UCR}_{y}$ circuit. Blue rectangles indicate groups of 3 CX gates which can be executed concurrently in the same cycle.

A straightforward circuit implementation of the $\text {UCR}_{y}$ gate in Eq. (3) consists of $2^{n_a}$ fully-controlled $R_y$ gates, where, for $i \in [2^{n_a}]$, the rotation angle is given by $\alpha _i$ and the $n_a$ address qubits are controlled on the state $\left| {i}\right\rangle$. An optimized circuit implementation existing of a depth-$2^{n_a}$ sequence of two-qubit CX gates and uncontrolled single-qubit $R_y$ rotations^17^ reduces the quantum resources^32^ at the cost of an increased classical overhead to solve the linear system

6 $$\begin{aligned} \theta _j = \sum _{i} W_{i,j}^{\prime } \alpha _i, \quad \text {for}\quad i,j \in [2^{n_a}], \end{aligned}$$

for the rotation angles $\vec {\theta }$. The angles $\theta _i$ are the parameters that are used in $R_y$ rotations of the compact circuit implementation for the $\text {UCR}_{y}$ gate in Fig. 1a. The linear system (6) is a *Walsh-Hadamard* transformation with Gray ordering, explained in more details in “Theoretical analysis of permuted $\text {UCR}_{y}$ gates” in the Supplementary Information. It can be solved efficiently classically in $\mathscr {O}(N \log N)$ operations through a *Fast Walsh-Hadamard Transform* (FWHT)^32^, where *N* denotes the length of the input sequence.

In another related work, we proposed FABLE^33^, which leverages compact $\text {UCR}_{y}$ and $\text {UCR}_z$ circuits to generate block-encodings of matrices, a widely used primitive in quantum linear algebra algorithms such as the quantum singular value transformation^34,35^.

Our new data encoding **QCrank** is an extension of the QPIXL–FRQI and MCRQI angle encodings. It uses parallel single-qubit rotations and CX gates acting on disjoint qubit pairs leading to much shorter circuits with a high degree of quantum parallelism. Our new **QBArt** basis encoding is a **QCrank** derivative that generates compact circuits for QIRs which use a basis encoding for the color mapping $\left| {c_i}\right\rangle$ such as NEQR. We do consider both **QCrank** and **QBArt** in the broader context of encoding *ordered data* $\vec {x}$ in a quantum state following Eq. (1), where the ordering of $\vec {x}$ is imposed by the tensor product of states on the address $\left| {i}\right\rangle$ and data qubits $\left| {c_i}\right\rangle$. Eq. (1) can be viewed as a generic case of a vectorized data structure with $\left| {i}\right\rangle$ the index and $\left| {c_i}\right\rangle$ encodes the value of $x_i$, respectively. This general quantum *index-value data structure* enables a natural representation and manipulation of different types of ordered data, such as DNA sequences, complex-valued series, 2D images, and time-ordered ECG waveforms, as shown by the experiments presented in the next section and in “Further experiments using QCrank and QBArt” in the Supplementary Information. The experiments leverage **QCrank**  and **QBArt**  based quantum algorithms and are executed on either NISQ^36^ hardware or noisy simulators. This work demonstrates that today’s NISQ devices can encode and compute on classical data sets. Because of the limitations on circuit depth that NISQ devices impose, we use relatively small data sets and simple processing tasks that are straightforward for classical computers. Nonetheless, our results showcase the limits of what is currently experimentally possible in this field. At the same time, our initial proof-of-concept results unveil the true possibilities for data analysis using quantum algorithms in the future as quantum hardware evolves.

## Results

In this section, we describe the main contributions of this paper, including the optimally scheduled parallel UCR gates, our new QROM encodings **QCrank** and **QBArt**, and a series of experiments with various types of data demonstrating their performance on real QPUs.

### Optimally scheduled parallel UCR gates

Figure 1a illustrates the standard compact circuit implementation for a $\text {UCR}_{y}$ gate^17^ with $n_a=5$ address qubits used in the QPIXL framework^32^. However, the $\text {UCR}_{y}$ circuit implementation is not unique. The positions of the control qubits of the CX gates can be permuted cyclically, as shown in Fig. 1b. We denote $\text {UCR}_{y}^{({n_a;s})}$ as the circuit implementation of a uniformly controlled $R_y$ rotation with $n_a$ address qubits and *cyclic permutations* $s \in [n_a]$. We show all 3 possible realizations of cyclic permuted UCRs for 3 address qubits, i.e., $s = 0 \rightarrow [0,1,2]$, $s = 1 \rightarrow [1,2,0]$, and $s = 2 \rightarrow [2,0,1]$. Note that the different implementations of the $\text {UCR}_{y}$ gate require a permutation of the linear system (6) to compute the rotation angles. However, the angles can always be computed with an $\mathscr {O}(N \log N)$ algorithm. More details are provided in “Theoretical analysis of permuted $\text {UCR}_{y}$ gates” in the the Supplementary Information.

The benefit of the permuted $\text {UCR}_{y}$ gates becomes clear when we combine multiple of them acting on different data qubits but sharing the same address qubits, as shown in Fig. 1c. In this case, the 1- and 2-qubit gates expressing the $\text {UCR}_{y}$ circuits with the 3 different permutations acting on 3 data qubits can be reordered to an equivalent circuit with the same critical depth as a single $\text {UCR}_{y}$ circuit operating on 1 data qubit. That is possible because the single-qubit $R_y$ rotations work on different qubits, and groups of CX gates act on different pairs of qubits. Consequently, both the $R_y$ and CX gates mutually commute and can be reordered to enable concurrent execution. We call this a *parallel uniformly controlled rotation* gate or $\text {p}\text {UCR}_{y}^{({n_a,n_d})}$ with $n_a$ address qubits and $n_d$ data qubits. The CX-depth of a $\text {p}\text {UCR}_{y}^{({n_a, n_d})}$ circuit is

7 $$\begin{aligned} d_{CX} \, {\, \le 2^{n_a} \, \lceil {n_d/\min (n_a, n_d)} \rceil }, ~~~ \text {for}~~ n_d,n_a>0. \end{aligned}$$

If $n_d \le n_a$, Eq. (7) is a strict equality, because the CX gates within a cycle act along edges in the bipartite connectivity graph shown on the right of  Fig. 1c that connect disjoint pairs of address and data qubits. Consequently, these CX gates can be executed in parallel on the quantum hardware, significantly shortening the execution time and improving the fidelity. If $n_d > n_a$, we require $n_d$ UCR circuits but only $n_a$ different UCR permutations exist. Following the gate grouping shown in Fig. 1c, we again find the CX depth on the right-hand side of Eq. (7). However, for certain ratios of $n_d/n_a$, we can commute independent CX and $R_y$ gates and regroup them in such a manner that further reduces the critical CX depth to the number of CX gates that are controlled by the address qubit with the most CX gates connected to it.

Table 1 illustrates the circuit depth advantage of the $\text {p}\text {UCR}_{y}$ gate implementation, introduced in Fig. 1c, over a logically equivalent serial implementation that does not use the cyclic permuted $\text {UCR}_{y}$ but instead just combines unpermuted $\text {UCR}_{y}^{({n_a;0})}$ circuits on different data lines. In this implementation, all the CX gates in a single cycle (blue box in Fig. 1c) share the same control qubit and cannot be executed concurrently on current quantum hardware. The circuit depths reported in Table 1 are taken *after* transpilation. We see that the Qiskit transpiler is not able to achieve the same data starting from the serial circuit compared to the parallel circuit. The reduction ranges between $2.1\times$ to $5\times$ for the size of circuits we considered. This maps 1-to-1 to a similar reduction in data loading time.

**Table 1:** Comparison of CX depth ($d_{CX}$) for parallel versus serial implementations of the UCR gate on $n_a = n_d \in \lbrace 4, 5, 6, 8, 10 \rbrace$ qubits.

$n_a$	(# address qubits)	4	5	6	8	10
$n_d$	(# data qubits)	4	5	6	8	10
$d_{CX}$	Parallel UCR	16	32	64	256	1025
	Serial UCR	32	81	193	1025	5121
	Reduction	$2.1\times$	$2.5\times$	$3.0\times$	$4.0\times$	$5.0\times$

A $\text {p}\text {UCR}_{y}^{({n_a, n_d})}(\vec {\alpha })$ gate implements the block diagonal unitary

8 $$\begin{aligned} \left[ \begin{array}{lll} R_y(\alpha _{0,0}) \otimes \cdots \otimes R_y(\alpha _{0,n_d-1}) &{} &{} \\ &{} \ddots &{} \\ &{} &{} R_y(\alpha _{2^{n_a}-1, 0})\otimes \cdots \otimes R_y(\alpha _{2^{n_a}-1, n_d-1}) \end{array}\right] , \end{aligned}$$

with $\vec {\alpha }= \left[ \alpha _{i,j} \right]$ a vector of $n_d \times 2^{n_a}$ rotation angles that encode the data $\vec {x}$.

Next, we describe our new QROM encodings **QCrank** and **QBArt**, which take advantage of this novel idea of optimally scheduled parallel UCR gates.

### QCrank data encoding

**Figure 2:**
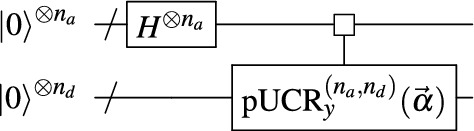
High-level block diagram of the **QCrank** circuit encoding $n_d \times 2^{n_a}$ real values $\vec {\alpha }$ on a state using $n_a+n_d$ qubits.

The quantum-parallel data encoding scheme we propose in this paper leverages the $\text {p}\text {UCR}_{y}$ circuits to generate an encoding. To this end, we only have to prepend *Hadamard* gates acting on the register of address qubits of the $\text {p}\text {UCR}_{y}$ circuit. That creates an equal superposition over all addresses as required for Eq. (1). Figure 2 shows the high-level block diagram of the **QCrank** circuit. We call our method **QCrank** as the arrangement of the CX gates in the $\text {p}\text {UCR}_{y}$ circuit diagram in Fig. 1c resembles a crankshaft in a combustion engine.

Similar to Eq. (4), it follows from Eq. (8) that the **QCrank** circuit prepares the state:

9 $$\begin{aligned} \begin{aligned} \left| {\psi _{\text {qcrank}}(\vec {\alpha })}\right\rangle&= \frac{1}{\sqrt{2^{n_a}}} \sum _{i=0}^{2^{n_a}-1} \left| {i}\right\rangle \otimes \left| {c_{i, 0}}\right\rangle \left| {c_{i, 1}}\right\rangle \cdots \left| {c_{i, n_d-1}}\right\rangle ,\\ \left| {c_{i, j}}\right\rangle&= \cos (\alpha _{i,j}/2) \left| {0}\right\rangle + \sin (\alpha _{i, j}/2)\left| {1}\right\rangle , \end{aligned} \end{aligned}$$

where $j \in [n_d]$. For a fixed index *i* and corresponding state $\left| {i}\right\rangle$ on the address qubits, the different rotation angles $\alpha _{i, 0}, \ldots , \alpha _{i, n_d-1}$ are encoded in the product state $|c_{i, 0} \rangle \cdots |c_{i, n_d-1} \rangle$. Consequently, the data recovery process can be decoupled into $n_d$ independent vectors of input parameters $\vec {\alpha }_{:, 0}, \ldots , \vec {\alpha }_{:, n_d-1}$, where $\vec {\alpha }_{:,j}$ denotes the vector constructed by taking all values with second index equal to *j*. By tracing out all data qubits except the *j*th, the FRQI state^23^ corresponding to the input parameter $\vec {\alpha }_{:,j}$ is retrieved. Formally,

10 $$\begin{aligned} \rho _\text {FRQI}(\vec {\alpha }_{:, j}) = {\textbf {Tr}}_{\mathscr {D}_{k: k \ne j}} \rho _{\text {qcrank}}(\vec {\alpha }), \end{aligned}$$

where $\mathscr {D}_{k: k \ne j}$ is the Hilbert space of all data qubits except the *j*th, and $\rho _\text {FRQI}$ and $\rho _{\text {qcrank}}$ are the density matrices defined in the usual manner. Eq. (10) provides a procedure to reduce the PDF on $n_a+n_d$ qubits measured from **QCrank** to $n_d$ PDFs on $n_a$ qubits for an FRQI encoding, for which we can recover the data using Eq. (5). As such, the state $\left| {\psi _{\text {qcrank}}(\vec {\alpha })}\right\rangle$ can be used to encode a dataset $\vec {x}$ of size $2^{n_a} \times n_d$ by mapping and/or rescaling real values $x_{i,j}$ to **QCrank** inputs $\alpha _{i,j} \in [0, \pi ]$. We note that **QCrank** uses shared address qubits with different data qubits, naturally resulting in shorter circuit depths as shown in Fig. 1. We remark that the **QCrank** state preparation defined in Eq. (9) is mathematically equivalent to the MCRQI encoding^29^.

In an idealized setting, **QCrank** allows for a lossless data encoding in a quantum state. In general, the purpose of the **QCrank** scheme is to enable potentially complex data processing or learning tasks on quantum computers. However, because of the limitations of current NISQ-era hardware, which is still severely restricted by gate infidelities, short coherence times, and cross-talk, we limit ourselves in this work to experimental demonstrations that either load and immediately recover the data through measurement, or apply a relatively simple and shallow data processing circuit.

For the task of data recovery, $\vec {x}^{~\text {meas}}$ can in principle be measured up to arbitrary precision with the error scaling as $N_s^{-1/2}$, where $N_s$ is the number of shots. For a NISQ device, we cannot expect that the reconstruction error decreases monotonically just by increasing $N_s$. For example, slight under- or over-rotation during the $R_y$ rotations on the data qubits can accumulate and distort the relationship between the *intended* and *achieved * rotation angles. Moreover, CX errors are typically an order of magnitude higher and lead to non-local errors. To compensate for all these effects, we introduce a hardware and circuit-dependent heuristic function $g(\cdot )$, called *adaptive calibration*,

11 $$\begin{aligned} \alpha ^{\text {meas}*}_i = g\left( \alpha ^{\text {meas}}_i \right) , \qquad i \in [2^{n_a}], \end{aligned}$$

which corrects the angles obtained from Eq. (5) to allow for a near-perfect **QCrank** decoding on NISQ devices. For clarity, we distinguish parameters recovered with the heuristic Eq. (11) by adding an asterisk as a superscript. The construction of $g(\cdot )$ from calibration measurements is discussed in more detail in Methods.

Additionally, for the **QCrank** experiments discussed in this work, we limit the input data $\vec {x}$ from continuous to discrete variables. We assume that $\vec {x}$ consists of a sequence of values drawn from a discrete set $[0, 1, \ldots , K-1]$. We call each possible input value a *symbol* and interpret *K* as the *max value* or $\lceil {\log _2(K)} \rceil$ as the *bit depth* in the case of digitized sequences, images, or time-series data. The advantage here is that to recover the discretized data $x^{\text {meas}*}$, we only need to be able to distinguish *K* different $\alpha ^{\text {meas}*}$ values that are spaced $\frac {\pi }{K}$ apart. In practice, we choose $K = 8$, which is small enough to simplify the task of distinguishing the *K* different rotation angles in the presence of noise and a limited measurement budget.

Despite all of the advantages of the **QCrank** encoding, we must point out that there are two difficulties in using it for large-scale data processing. *First*, to decode the data from a **QCrank** encoding relies on accurately measuring the PDF to compute the data using Eq. (5), which clearly scales exponentially with the number of address qubits $n_a$. For example, assuming 8 address qubits and 16 data qubits, **QCrank** can store $2^8 \times 16 = 2^{12}$ real input values on a QPU using 24 qubits. To recover all $2^{12}$ real values, we need to accurately measure probabilities for all $2^8=256$ address bit-strings separately for each data qubit. Assuming we aim for an error of 1% for probabilities used in Eq. (5), it would require about $10^4$ shots per bit-string. Hence, about $10^6$ shots would be required to recover 100s of stored values with a desired precision of 1%. This estimate on the number of shots is based purely on sampling error. It does not consider the infidelity of the actual quantum hardware, which will further increase the necessary amount of shots. Using **QCrank** in this manner can be practical if we plan to recover only a small number of values from the QPU, in which case specific address bits can be queried on the address register. Furthermore, merely encoding and decoding classical data on a QPU is of limited interest beyond benchmarking and verification purposes. Consequently, some quantum data processing that condenses the information from the high-dimensional input space to a low-dimensional *solution* must be applied on the QPU before we read it out classically. *Second,* it is not trivial to develop data processing algorithms that act on the angle encoding used in **QCrank** and return a condensed result. To overcome these issues, we propose the **QBArt** encoding, which uses the NEQR *basis encoding*.

### QBArt data encoding

**Figure 3:**
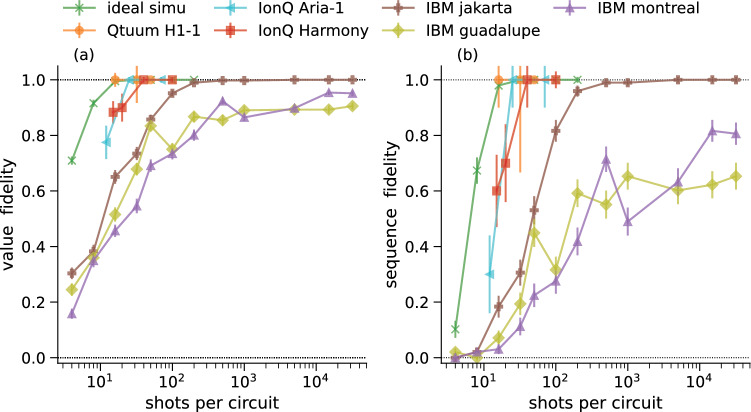
Reconstruction fidelity for the **QBArt** benchmark experiment that uses 4 4-bit integers loaded on 6 qubits. Data is shown for Quantinuum, IonQ, and IBMQ QPUs: (**a**) reconstructed value fidelity (**b**) reconstructed sequence fidelity. The H1-1 experiment with 50 shots was only performed for one input sequence, so no error bars are shown.

Our *Quantum Binary representation Arithmetic* (**QBArt**) encoding retains the quantum-parallel feature of **QCrank** while encoding the data in the well-studied basis encoding as used in NEQR^26^. Formally, **QBArt** generates circuits with identical structure as the **QCrank** circuit (Fig. 2), except that the rotation angles are now restricted to two discrete values $\vec {\alpha }\in \lbrace 0, \pi \rbrace$. It was noted in Figure 3 and Definition 6 in our previous work^32^ that serial $\text {UCR}_{y}$ gates can be used to prepare a NEQR state. **QBArt** instead leverages the compact parallel UCR circuits to efficiently prepare the NEQR state on real QPUs.

**QBArt** offers a lower density of information storage than **QCrank** because data qubits now hold only superposition of $\lbrace 0,1\rbrace$’s instead of superposition of real numbers encoded as $R_y$ rotations. However, the output of **QBArt** is sparse, so decoding requires a far smaller number of shots. Furthermore, instead of relying on estimating the PDF and using Eq. (5) to recover the data, the observed bit-strings themselves contain the data. Theoretically, that leads to an exact data value reconstruction using a single observation. We will experimentally demonstrate that post-processing by *majority voting* suppresses the noise artifacts very effectively for **QBArt** executed on NISQ hardware. The number of data qubits used in **QBArt** sets the absolute precision with which a quantum computation is performed on the data, regardless of the QPU’s fidelity. Since many data processing tasks require 8 to 16 bits of precision, this is a manageable overhead for the qubit count, even for existing QPUs.

### **QBArt** benchmarking

In this section, we run a benchmark using a **QBArt** circuit of fixed size on various available QPUs. We consider this to be an application-level benchmark^37,38^ that specifically evaluates the performance of the QPU to serve as QROM. We use this benchmark to inform our choice of QPU to run the data processing experiments described in Quantum Data Processing on H1-1.

We use a **QBArt** circuit with 2 address and 4 data qubits such that it can be executed on any QPU with at least 6 qubits. A circuit of this size can load 4 bit strings, each of length 4, and we use randomly generated bit strings as input data. Our benchmarks are executed on Quantinuum, IonQ, and IBMQ QPUs. The specifications of these different backends are summarized in Table 2.

**Table 2:** Basic characteristics of the QPUs used for benchmarking. The listed average CX error is officially reported by the hardware providers at the time of circuit execution.

	Qubits	CX error	CX depth	# runs
Quantinuum H1-1	20	0.003	8	1–10
IonQ Harmony	11	0.040	8	10–15
IonQ Aria	23	0.004	8	10
IBM guadalupe	16	0.012	42	98
IBM montreal	27	0.020	39	98
IBM jakarta	7	0.008	42	98

A **QBArt** encoding generates the optimal circuit for QPUs on which a bipartite qubit connectivity is naturally available. The trapped ion QPUs (H1-1, Harmony, and Aria) natively allow such connectivity. However, for the transmon-based QPUs from IBMQ with *heavy-hexagonal* connectivity, the transpiled circuits become deeper due to the inevitable swap operations (see Table 2). Before transpilation, the circuit depth is 8 CX-cycles. We also run the same experiment on the noise-free Qiskit simulator for reference.

We compare shot dependence of two metrics pertaining to (1) the fidelity of individual values in the sequence and (2) the whole sequence being recovered correctly, both defined in Methods. The results are shown in Fig. 3. The trapped-ion QPUs require only 100 shots to recover the entire sequence and significantly outperform the transmon-based QPUs. The two decisive factors are differences in the fidelity of entangling gates and the versatility of the native connectivity. Only on the IBM *jakarta*, which has a relatively low CX-gate error, can we achieve 100% sequence fidelity but at the expense of more than 2000 shots. It requires 100 times more shots compared to an ideal noise-free QPU.

The Quantinuum H1-1 QPU achieves the highest fidelity among all the QPUs tested and will be the backend we use for the data processing experiments in the next section.

### Quantum data processing on H1-1

In the following, we describe four experiments using different types of data executed on H1-1 as summarized in Table 3.

**Table 3:** **QCrank** and **QBArt** data processing experiments executed on Quantinuum H1-1.

	Experiment #1	Experiment #2	Experiment #3	Experiment #4
Encoding	**QBArt**	**QBArt**	**QBArt**	**QCrank**
Data type	DNA Sequence		Time-series	Binary image
Objective	DNA match	Hamming weight	Complex conjugate	LBL logo I/O
QPU	H1-1	H1-1	H1-1	H1-1
Addr. qubits	4	4	5	4
Data qubits	12	3	10	8
Ancillas	–	1	–	–
Reset ops	5	–	–	–
Input (bits)	192	48	320	384^⋆^

^⋆^Assuming 3-bit resolution per real value encoded by **QCrank**

#### DNA sequences

The genetic code of any organism is described as a sequence of *codons* that encode specific amino acids. A codon consists of three nucleotides. Since 4 types of nucleotides exist in nature (*A*, *T*, *G*, *C*), there are 64 different codons, which is equivalent to 6 classical bits of information. On a quantum computer, we will use 6 qubits to encode a codon by assigning 2-qubit basis states to the 4 nucleotides,

12 $$\begin{aligned} A&= \left| {00}\right\rangle , T = \left| {01}\right\rangle , G = \left| {10}\right\rangle , C = \left| {11}\right\rangle , \end{aligned}$$

and constructing the tensor product of 3 2-qubit states, e.g., $ACT = \left| {001101}\right\rangle$ or $ATG = \left| {000110}\right\rangle$. To compare 2 DNA sequences made of codons, we compute on pairs of codons, requiring 12 qubits in total. Consequently, the integer values in Eq. (1), $c_i \in [4096]$, allow the encoding of 2 codons as a quantum state $\left| {a_0 \cdots a_5 \, b_0 \cdots b_5}\right\rangle$, being again a tensor product of the quantum states of 2 codons.

We realize that the quantum DNA data processing primitives presented in this section are toy models based on relatively small data sets and do not provide computational advantage over far more efficient conventional algorithms. Our goal is rather to demonstrate what is possible with current generation quantum computers and motivate future work in this field.

##### Pattern matching

**Figure 4:**
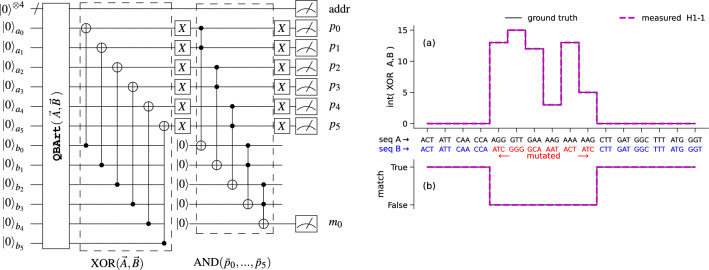
(**Left**) Quantum circuit computing the match between two 16 elements long input sequence of 6-bit values A and B, encoded on qubits $a_i$ and $b_i$, respectively. The **QBArt** unitary prepares the initial state. The 6 CX gates compute the XOR between bits of sequences element. The following 5 nested Toffoli gates set the output qubit $m_0$ to state $\left| {1}\right\rangle$ if both 6-bit input pairs match. The intermediate XOR output result is also measured on qubits $p_i$. (**Right**) Results obtained by the DNA sequence matching executed on Quantinuum H1-1 QPU. The algorithm correctly detects the differences between the 6 codons in positions 5 to 10, marked in red. (**a**) 6-bit XOR$(\vec A, \vec B)$ output sequence and (**b**) measured match-bit, both follow the ground-truth.

In *Experiment #1*, the inputs are two codon sequences of equal length, and the output is a 1-bit sequence of the same length that contains 1 for every position where the codons match and 0 elsewhere. The additional 6-bit wide output sequence encodes which nucleotides did not match. The **QBArt** circuit (Fig. 4(Left)) implementing *Experiment #1* requires a total of 16 qubits, 4 of which are used as address qubits, and the 12 data qubits encode the codons of both sequences using 6 bits each. To save on quantum resources, we ‘recycle’ 5 qubits in the middle of the circuit by applying a *reset* gate, which is available on most QPUs. The total circuit depth is 68 CX-gates, of which 48 are needed by **QBArt** itself, following Eq. (7). We perform this DNA matching experiment on the real 20-qubit trapped-ion QPU from Quantinuum, H1-1. The input sequence A is a random snippet from the COVID-19 genome strain^39^. Sequence B is a copy of A, but the 6 codons in positions 5 to 10 are randomly altered, as shown in Fig. 4(Right). At the expense of 600 shots and using the majority voting technique, we achieve an exact result for all 16 codon pairs. The results shown in Fig. 4 (Right, (a)) confirm that the 6 XOR bits $p_i$ are all 0 when the two codons match, and some are non-zero otherwise, following the ground-truth. The bit $m_0$, indicating a match, is also correctly computed, as shown in Fig. 4(Right, (b)).

##### Hamming weight computation

**Figure 5:**
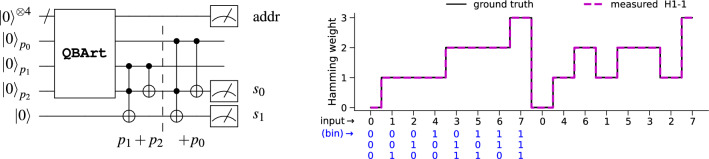
(**Left**) Quantum circuit computing the Hamming weights (HW) for a sequence of 16 3-bit integers. **QBArt** unitary encodes the input sequence on qubits $p_i$. The following 1st sub-ciruit computes the partial HW for inputs $p_1,p_2$. The 2nd one adds the value of $p_0$ to the partial HW stored binary on qubits $s_0,s_1$. The final HWs are measured on qubits $s_0,s_1$, for all addresses. (**Right**) Results of Hamming weight computation for a sequence of 16 3-bit integers, executed on Quantinuum H1-1.

The *Hamming distance* between two bit-strings tells us in how many places they differ from one another; hence, when applied to codon sequences, it is an essential tool for studying the evolution of the genetic code. The *Hamming weight* of a binary string is defined as the number of bits set to 1. In *Experiment #1* we have already computed the binary XOR value between the two codons, stored at $p_i$. We will now pass them to the Hamming weight algorithm to compute the desired Hamming distance. *Experiment #2* computes the Hamming weights for a 3-bit sequence of length 16. We use **QBArt** with 4 address qubits and 3 data qubits to encode the input and 1 *ancilla* qubit. The complete **QBArt** circuit (Fig. 5 (Left)) uses 8 qubits, has a CX-depth of 28 cycles on an *all-to-all* connected QPU. The experimental results from H1-1 for a pseudo-random input sequence and using 300 shots agree with the ground-truth *exactly*, as shown in Fig. 5(Right).

We recognize that the quantum circuit in Fig. 5 (Left) does only ‘half’ of the job since it reduces only 3 inputs $p_0,p_1,p_2$ to 2 output bits $s_0,s_1$. However, with enough resources, we can add a second copy of the same circuit acting in parallel on the output qubits $p_3,p_4,p_5$ from the circuit shown in Fig. 4 (Left) and compute the missing 2nd Hamming weight, to be stored on qubits $s_2,s_3$. Then, we can apply a binary adder on two 2-bit inputs, using one of the known quantum circuits^40^, to obtain the total Hamming distance between the 2 6-bit codons.

#### Complex conjugate

**Figure 6:**
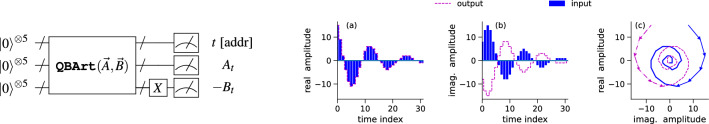
(**Left**) **QBArt** circuit for *Experiment* *#3* computing the complex conjugate of the input sequence $A_{t} +j B_{t} \rightarrow A_{t} -j B_{t}$ The values of $\vec {A}$ and -$\vec {B}$ are retrieved as *signed int* at the addresses encoded as *unsigned int*. (**Right**) Results of the complex conjugate on complex-valued time-series obtained on the H1-1 QPU are presented in magenta. The input is shown in blue. (**a**) and (**b**) show real and imaginary components of the pendulum amplitude, respectively. (**c**) depicts its trajectory as function of time. The conjugation operation inverts the sign of the imaginary component.

The time evolution of a damped pendulum described by Eq. (13) is an example of a process described by the ordinary differential equation, ODE, in the complex domain. The real and imaginary components of the complex-valued amplitude *C*(*t*) are denoted as *A*(*t*) and *B*(*t*), respectively. They are plotted independently as a parametric trajectory in blue on 3 panels in Fig. 6 (Right). The purpose of *Experiment #3* is to store the time-series $C_t$ on the QPU, compute the complex conjugate of the amplitude, $C_t^*$, and recover the resulting new time-series through measurement.

13 $$\begin{aligned} C_t&= a \exp {\left[ (b +j c) t +c\right] }, ~~~ C_t \in \mathbb {C},~~~\text {for}~~ t \in [0,\ldots ,31]\nonumber \\ A_t&= {\textbf {Re}}(C_t),\nonumber \\ B_t&= {\textbf {Im}}(C_t), \end{aligned}$$

where $j=\sqrt{-1}$ and the real parameters *a*, *b*, *c*, *d* are conveniently chosen to match the initial condition $\left|{A_0}\right|,\left|{B_0}\right| \simeq 2^4$ and $\left|{A_{31}}\right|,\left|{B_{31}}\right| \simeq 1$.

We use the *signed integer* 5-bit representation for both components $A_t$ and $B_t$, stacked as a single 10-bit input for the **QBArt** circuit (Fig. 6 (Left)). Next, we invert the sign of the $B_t$-data and perform the measurement. This circuit uses 5 address qubits and 10 data qubits, with a CX-depth of 64. Only one cycle of *X* gates is needed to invert all 32 $B_t$ values stored in the Hilbert space. The output $-B_t$ values are *1’s complementary* and need to be decoded as such classically, back to *signed* integers. This method of computing 1’s complementary was proposed in the original NEQR paper^26^. However, to the best of our knowledge, we present the first practical demonstration of such computation using a real QPU. To recover all 32 10-bit values *exactly*, the H1-1 requires $10^3$ shots.

#### 2D image

**Figure 7:**
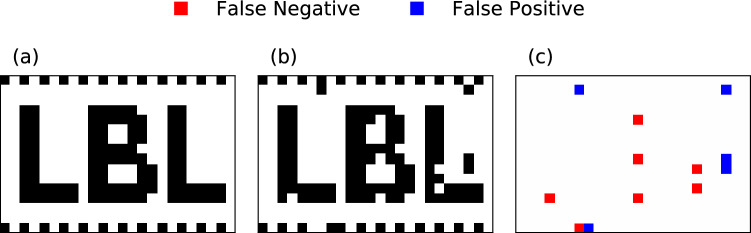
Demonstration of recovery of a black and white 384 pixels image using **QCrank** *Experiment #4* executed on the Quantinuum H1-1 real QPU. (**a**) ground-truth image, (**b**) recovered image has 97% of correct pixels, (**c**) residual showing the locations of 12 incorrect pixels.

Any multi-dimensional indexed dataset can always be enumerated as a 1-dimensional sequence. Therefore, our proposed quantum data encodings can directly encode 2D images as well. *Experiment #4* demonstrates the **QCrank** encoding of the black and white image of size 384 bits shown in Fig. 7a. The recovered image from the Quantinuum H1-1 QPU is shown in Fig. 7b. At the expense of 7000 shots, **QCrank** recovers 97% of the pixels correctly. The location of the 12 incorrect bits is random, as shown in Fig. 7c. This experiment shows that we can store, today, a non-trivial sized image on a 12 qubit system. Moreover, an image encoded with **QBArt** could be manipulated by a quantum algorithm, such as filtering^13^.

#### Simulations

In addition to the four experiments executed on H1-1, we explore the robustness and versatility of our proposed encodings with additional simulated experiments presented in “Further experiments using QCrank and QBArt” in the Supplementary Information. In particular, we demonstrate the feasibility of storing and retrieving an arbitrary waveform on a QPU. We generate a synthetic electrocardiogram (ECG) time-series of length 64, digitize it with the 6-bit resolution, and encode it using **QBArt**. We also study the dynamic range and recovery fidelity for **QCrank** as a function of the simulated noise and the number of shots.

## Discussion

This work presents two significant contributions to practical realization of quantum data encoding and analysis. First, the parallel uniformly controlled rotation circuits (pUCR) significantly reduce the critical circuit depth ($d_{CX}$) compared to a serial implementation. The pUCR circuit leverages concurrent execution of CX gates which leads to compact circuits that are well-suited for QPUs with a high degree of connectivity, such as those based on the ion trap technology. Second, we propose two data encoding methods that use pUCR circuits: **QCrank**  which encodes continuous real data as rotation angles, and **QBArt**  which encodes integer data in binary representation. Finally, we present an extensive collection of experiments conducted on different real QPUs with both **QCrank** and **QBArt** encodings. We demonstrate successful quantum data encoding and analysis at a considerably larger scale than achieved in previous studies. This is enabled by two new error mitigation strategies for **QCrank** and **QBArt**  respectively, to correct the noisy hardware results.

Our experiments show that the Quantinuum H1-1 QPU can reliably prepare a **QCrank** state that encodes 384 black-and-white pixels on 12 qubits. We introduce an adaptive calibration routine to compensate for the hardware noise and achieve a 97% recovery fidelity using 7,000 shots. This **QCrank** state on $n_a = 4$, $n_d = 8$ qubits encodes 128 real values. It is 16$\times$ more then the state-of-the-art QML experiments in^9^, which extracted from the MNIST images the 8-dimensional real feature vectors in order to encode them on quantum hardware. The data encoding method in^9^ allow for trade-offs between ancillary qubits and circuit depth. It is an interesting topic of future research to investigate if our circuits can allow for a similar trade-off.

Our experiments with **QBArt** show that the H1-1 QPU can, with near-perfect fidelity, (1) simultaneously encode two DNA sequences of 16 codons on 16 qubits, using 6 bits per codon, and compute the positions where the sequences are mismatched, (2) compute the Hamming weight of a sequence of 16 3-bit integers, and (3) compute the complex conjugate of a sequence of 32 complex values with real and imaginary parts both encoded with bit-depth 5. Furthermore, we successfully use a majority voting technique to reliably identify the correct results from the measured bit strings. Finally, we report the results of a 6 qubits **QBArt** encoding benchmark comparing the recovered value and sequence fidelity on Quantinuum, IonQ, and IBM QPUs. This experiment highlights the superiority of ion trap QPUs over superconducting QPUs for preparing a **QBArt** state. That is partially attributed to the qubits connectivity: additional swap gates are required to run a pUCR circuit on superconducting QPUs.

For a fixed number of qubits, the angle encoding in **QCrank** and FRQI^24^ can store more data than a binary encoding in **QBArt** and NEQR^26^. However, data processing of angle encodings is considerably more complex than computing on binary encodings where classical binary logic operations can be efficiently converted to reversible quantum operations^1^. Additionally, our experiments show that binary data can be recovered with greater fidelity and using an order of magnitude fewer shots compared to data stored in an angle encoding.

Our multiplexor pUCR gates are closely related to circuit implementations used for QROM/QRAM. For example, the implementation of the bucket brigade QRAM^18^ leverages parallel decompositions of commuting Toffoli gates which closely resemble the pUCR circuit for the special case $n_a = n_d = 2$. It is important to note, however, that our circuits have been designed for execution in the current NISQ era, whereas QRAM implementations typically target future fault-tolerant architectures. It is likely that our pUCR circuits are suboptimal in a fault-tolerant setting because the logical synthesis of the $R_y$ gates will lead to a large overhead compared to^18^.

One exciting topic for future study that our results hint at is the potential of the compact, parallel **QCrank** circuits in the context of hybrid algorithms such as Variational Quantum Algorithms (VQA)^41^ or Quantum Machine Learning (QML)^42^ tasks. In this context, the rotation angles in the **QCrank** circuit are considered free parameters that are variationally optimized in a quantum-classical hybrid iteration where the cost function is evaluated on the QPU.

## Methods

### Metrics of fidelity

We define three metrics to characterize the quality of the recovered results using **QCrank** and **QBArt** encodings on noisy QPUs:

- **Dynamic range ** ($D_r$) is defined as the distance between the expectation values of the reconstructed angle $\alpha ^{meas}$ (Eq. 5) for the first and last symbol. It is applicable only for **QCrank** with input quantized into *K* symbols $a_0,\ldots ,a_{K-1}$
  14 $$\begin{aligned} D_r = \frac{\mathbb {E} \big ( \alpha ^{meas} ( a_{K-1}) \big ) - \mathbb {E} \big ( \alpha ^{meas} ( a_{0}) \big )}{\alpha ( a_{K-1}) - \alpha ( a_{0})}. \end{aligned}$$
  The domain of $D_r$ is [0, 1], where 1 corresponds to a perfect result and 0 means that pure noise is measured.
- **Recovered value fidelity** (RVF) is defined as the probability to recover the correct symbol at a given position in the sequence, averaged over the sequence.
- **Recovered sequence fidelity** (RSF) is defined as the probability of all recovered values in a sequence being correct. In the simplest case, $\text {RSF}\sim (\text {RVF})^N$, with *N* being the length of the sequence.

### Adaptive calibration for QCrank

Based on experiments using a noisy simulator, we observed that the dynamic range ($D_r$) is reduced with increasing gate infidelity, which leads to incorrect symbol reconstruction, as shown in Supplementary Fig. S1. Therefore, we developed an *adaptive calibration* method to compensate for these distortions. For each input symbol, $a_i$, we measure the average reconstructed output $\mathbb {E} (\alpha ^{meas}_i$). We then define a lookup table with $K-1$ thresholds $\tau _i$,

15 $$\begin{aligned} \tau _i= \frac{ \mathbb {E} (\alpha ^{meas}_i) + \mathbb {E} (\alpha ^{meas}_{i+1})}{2}, \end{aligned}$$

set halfway between those *K* averages, shown as horizontal dotted lines in Supplementary Fig. S1. Next, we reanalyze the same experiment and assign the discrete reconstructed symbols using a heuristic function $g(.), ~g: \alpha ^{meas} \rightarrow a^{meas*}$ defined by the following lookup table:

16 $$\begin{aligned} g(\alpha ^{meas})= {\left\{ \begin{array}{ll} a_0,&{} \text {if }~ \alpha ^{meas} < \tau _0\\ a_{K-1},&{} \text {if }~ \alpha ^{meas} \ge \tau _{m-2}\\ a_j, &{} \text {if } ~\alpha ^{meas} \in [ \tau _{j},\tau _{j+1}) \end{array}\right. }. \end{aligned}$$

### Majority voting for QBArt

For measurements subject to a low noise level, only a few of the measured bits will be incorrect, resulting in either corrupted address or corrupted data bits. To compensate for this uncorrelated noise, for each measured address, we choose the reconstructed data to be the most probable value (MPV) of all the data sub-strings collected from the measured bit-strings. This procedure is equivalent to majority voting on the data bit-strings. As the errors on the address bit-strings manifest in the same way, MPV selection also suppresses it.

### Supplementary Information

Supplementary Information.

## Data Availability

The data generated for the **QCrank** and **QBArt** experiments can be found at https://github.com/QuantumComputingLab/qpixlpp/tree/master/examples/SciRep2024_QBArt_QCrank_Data.
