# Supplementary material for: Quantum-parallel vectorized data encodings and computations on trapped-ion and transmon QPUs
Source: Sci Rep. 2024 Feb 10;14:3435. doi: 10.1038/s41598-024-53720-x (PMC10858957; doi:10.1038/s41598-024-53720-x)
Supplement: Supplementary file 1 — Supplementary Information. [file 41598_2024_53720_MOESM1_ESM.pdf]

Supplementary information for “Quantum-parallel vectorized data encodings and computations on trapped-ion and transmon QPUs”

Jan Balewski[1], Mercy G. Amankwah[1,2], Roel Van Beeumen[3], E. Wes Bethel[4], Talita Perciano[5,*], and Daan Camps[1,*]

[1]National Energy Research Scientific Computing Center, Lawrence Berkeley National Laboratory, Berkeley, CA 94720, USA
[2]Department of Mathematics, Applied Mathematics and Statistics, Case Western Reserve University, Cleveland, OH 44106, USA
[3]Applied Mathematics and Computational Research Division, Lawrence Berkeley National Laboratory, Berkeley, CA 94720, USA
[4]Computer Science Department, San Francisco State University, San Francisco, CA 94132, USA
[5]Scientific Data Division, Lawrence Berkeley National Laboratory, Berkeley, CA 94720, USA
[*]Corresponding authors: tperciano@lbl.gov, dcamps@lbl.gov

Theoretical analysis of permuted UCR$_y$ gates

In this section, we provide a more detailed analysis of the construction of UCR$_y$ and the cyclically permuted UCR$_y$ circuits, illustrated in Fig. 1 in the Main Text. The construction of regular UCR$_y$ circuits is well-understood[1,2], but we include a brief discussion about these concepts for the sake of completeness. The cyclically permuted UCR$_y$ and corresponding parallel UCR$_y$ circuits are a novel contribution to the best of our knowledge.

Throughout this text, two elementary properties of Pauli-Y rotations will prove to be useful:

$$\begin{aligned} \text{angle addition:} &\quad R_y(\phi_0)R_y(\phi_1) = R_y(\phi_0+\phi_1), \\ \text{angle negation:} &\quad XR_y(\phi)X = R_y(-\phi). \end{aligned} \tag{1}$$

Motivating example

We start with the case of a single address qubit as the simplest possible case to motivate the usage of the compact UCR$_y(\alpha)$ circuits. Using the decomposition for a singly-controlled R_y gate into CX and single-qubit gates[3],

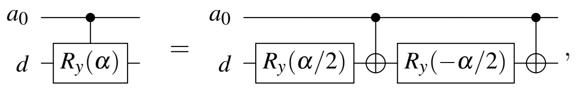

, (2)

which directly follows from the properties Eq. (1). We can decompose a UCR$_y$ circuit with a single address qubit as follows:

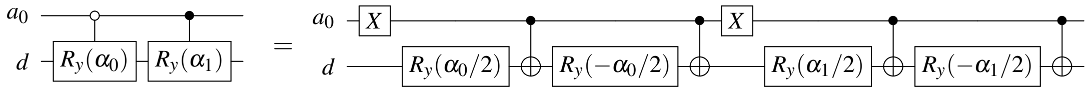

. (3)

This decomposition is clearly suboptimal as it is known that any two-qubit gate can be decomposed in a circuit with at most 3 CX gates[4]. Using a compact UCR$_y$ circuit, we can implement the circuit using two CX gates only:

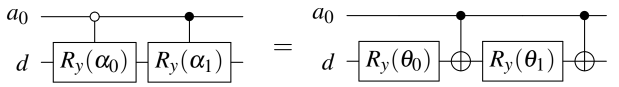

, with $\begin{aligned} \alpha_0 &= \theta_0 + \theta_1 \\ \alpha_1 &= \theta_0 - \theta_1 \end{aligned}$. (4)

Using Eq. (1), we can verify that Eq. (4) holds by considering the action of the circuit for the two possible basis states of the address qubit:

- If the address (control) qubit is in the $|0\rangle$ state, the circuit on the left applies a $R_y(\alpha_0)$ rotation to the second qubit. The circuit on the right applies $R_y(\theta_0)R_y(\theta_1) = R_y(\theta_0+\theta_1)$ to the second qubit, which is equivalent if $\alpha_0 = \theta_0+\theta_1$.
- If the address (control) qubit is in the $|1\rangle$ state, the circuit on the left applies a $R_y(\alpha_0)$ rotation to the second qubit. The circuit on the right applies $R_y(\theta_0)XR_y(\theta_1)X = R_y(\theta_0)R_y(-\theta_1) = R_y(\theta_0-\theta_1)$ to the second qubit, which is equivalent if $\alpha_1 = \theta_0-\theta_1$.

The relation between α_0, α_1 and θ_0, θ_1 is the Walsh-Hadamard transformation (Eq. (6) in the Main Text) of dimension 2.

Cyclically permuted UCR$_y$ circuits.

In the case of a single address qubit controlling UCR$_y$, $(n_a = 1)$, the decomposition Eq. (4) is *unique*. For $n_a > 1$, there are n_a different realizations of the UCR$_y$ circuit that cyclically permute the index of the control qubit of the CX gates. We illustrate this idea for $n_a = 2$. The first realization UCR$_y^{(2;0)}$, with the *permutation shift* $s = 0$ added as the 2nd superscript. For $s = 0$, we have the natural ordering of the address qubits as $[0,1]$:

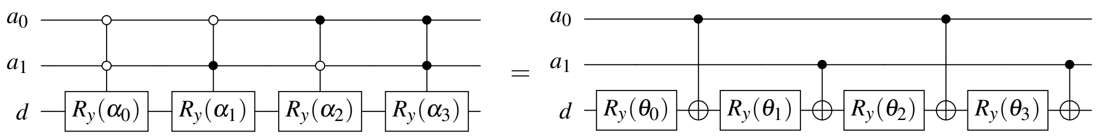

with

$$\alpha_0 = \theta_0 + \theta_1 + \theta_0 + \theta_1,$$
$$\alpha_1 = \theta_0 + \theta_1 - \theta_0 - \theta_1,$$
$$\alpha_2 = \theta_0 - \theta_1 - \theta_0 + \theta_1,$$
$$\alpha_3 = \theta_0 - \theta_1 + \theta_0 - \theta_1.$$

The linear system relating α_i's to θ_j's can again be derived using Eq. (1). The second realization UCR$_y^{(2;1)}$ cyclically permutes the position of the control of the *CX* gates by 1, i.e., $s = 1$ which leads to the ordering of the address qubits as $[1,0]$:

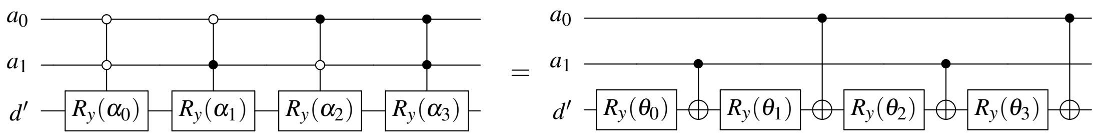

with

$$\alpha_0 = \theta_0 + \theta_1 + \theta_0 + \theta_1,$$
$$\alpha_1 = \theta_0 - \theta_1 - \theta_0 + \theta_1,$$
$$\alpha_2 = \theta_0 + \theta_1 - \theta_0 - \theta_1,$$
$$\alpha_3 = \theta_0 - \theta_1 + \theta_0 - \theta_1.$$

The only difference in the $s = 1$ linear system that relates α_i's to θ_j's are the signs that are highlighted in gray. This is essentially a permutation of the linear system for $s = 1$ that can be computed efficiently by computing the position of the bit where two consecutive Gray code $\mathtt{g}_i$ and $\mathtt{g}_{i+1}$ differ[1], where $\mathtt{g}_i$ is the reflected binary Gray code of the integer i.

This approach generalizes to any $n_a > 0$ address (control) qubits. There always exist n_a different decompositions of the UCR$_y$ gate using the approach outlined above. The parameters θ_j can be computed from angles α_i using the *Fast Walsh-Hadamard Transform* (FWHT)[2] followed by a permutation that depends on the shift s. The classical complexity of this algorithm is $\mathcal{O}(n_a 2^{n_a})$. As a pUCR$_y$ gate with n_d data and n_a address qubits is equivalent to n_d permuted UCR$_y$ gates with n_a address qubits, the cost of classical data preprocessing for **`QCrank`** and **`QBArt`** thus is $\mathcal{O}(n_d n_a 2^{n_a})$.

Further experiments using QCrank and QBArt

In addition to the four experiments on real hardware, as presented in "Quantum data processing on H1-1" in the Main Text, we performed five other experiments using both **`QCrank`** and **`QBArt`** using noise-free and noisy circuit simulators. These additional experiments explore the robustness and versatility of the proposed encodings and recovery techniques. General information and setup for the experiments shown next are summarized in Supplementary Table S1. We use the same three metrics of fidelity as in "Metrics of fidelity" in the Main Text: dynamic range (D_r), recovered value fidelity (RVF), and recovered sequence fidelity (RSF). These metrics, along with our new *adaptive calibration* method, are described in "Methods" in the Main Text.

We use `Qiskit-Aer` as the primary circuit simulator with four custom noise models, listed in Supplementary Table S2, emulating the finite fidelities and coherence times of the QPUs that we used in the earlier experiments: (a) **noise-free** ideal simulator for circuit validation and probing ideal performance, (b) **minimal noise** levels to study the impact of introducing some noise over the ideal results, (c) **H1-proxy** noise model that approximates the bit-flip and thermal noise present on the H1-1 Quantinuum trapped-ion QPU, (d)

Experiment	#5 **QCrank**	#6 **QCrank**	#7 **QCrank**	#8 **QBArt**	#9 **QBArt**
Data type	Integer sequence				Time series
Objective	Test D_r, RVF, RSF				ECG wave I/O
Simulator	noisy† `Qiskit-Aer`				Quantinuum H1-1E‡
addr. qubits (n_a)	4	4	2 - 7	5	6
data qubits (n_d)	8	8	8	10	6
# addresses	16	16	4 - 128	32	64
input (bits)	384	384	96 - 3,000	320	384

†`Qiskit` simulator with noise level tuned to approximate real QPUs.

‡Quantinuum proprietary simulator H1-1E is tuned to emulate H1-1.

Supplementary Table S1. Summary of simulated experiments using **`QCrank`** and **`QBArt`** to provide further insights about the proposed encodings.

IBMQ-proxy noise model based on the IBMQ transmon QPUs. The `Qiskit-Aer` simulator is set up to use *all-to-all* connectivity, which is not valid for IBMQ QPUs, and assumes no limit on the gates multiplicity per cycle, which is not valid for H1-1. Consequently, the CX-depth of the simulated circuits are shorter in comparison to the real hardware execution, as SWAPs are not required, and the fidelities retrieved from our simulations are better compared to the respective QPUs.

**Noise model**	ideal	minimal	H1-proxy	IBMQ-proxy
**Objective**	circuit verification	fidelity at low noise	fidelity of hardware	
SPAM error♣	0	$1 \cdot 10^{-3}$	$3 \cdot 10^{-3}$	$2.5 \cdot 10^{-2}$
U3 error	0	$1 \cdot 10^{-3}$	$5 \cdot 10^{-5}$	$4.0 \cdot 10^{-4}$
CX error	0	$1 \cdot 10^{-3}$	$3 \cdot 10^{-3}$	$1.4 \cdot 10^{-2}$
duration T1/U3♠	∞	$1 \cdot 10^{5}$	5000	2000
duration T1/CX	∞	$1 \cdot 10^{5}$	170	200
RVF	1.0	0.78	0.68	0.26
D_r	0.99	0.90	0.67	0.29

♣bit flip probability error

♠thermal noise model relies only the ratio of coherence time to gates duration.

Supplementary Table S2. Noise model configurations used for simulated experiments listed in Supplementary Table S1.

Adaptive calibration procedure for QCrank (Experiment #5)

The distortion correction function, $g(\cdot)$, that was introduced in Eq. (11) in the Main Text, is a heuristic calibration that can be applied to the raw **`QCrank`** data obtained from the QPU in order to improve the recovery fidelity compared to directly applying Eq. (5) in the Main Text to the raw measurements. The goal of this experiment is to study the performance of the adaptive calibration for the different noise models listed in Supplementary Table S2. We use a **`QCrank`** circuit with 4 address- and 8 data-qubits such that the circuit has a depth of 32 CX-cycles and can load $2^4 \times 8 = 128$ real values. We generate 98 random input data sequences of length 128 with values selected out of 8 different symbols, i.e, a bit-depth of 3. The total capacity of this circuit configuration is 384 classical bits of information. Each **`QCrank`** circuit is measured for $3 \cdot 10^3$ shots for each noise model and every data set.

Supplementary Fig. S1 shows the distribution of the angles α^{meas} (reconstructed using Eq. (5) in the Main Text based on $3 \cdot 10^3$ shots obtained from the four different simulators) as a function of the input symbol. The reconstructed angles are visualized using *violin plots* that show the distribution of the measured angles for each different input symbol. The average angle is indicated by the short blue bar. We observe that, for the ideal noise-free simulator, the measured averages line up exactly with the input angles that are shown as red dashed lines. Similarly, the recovered angles will have no systematic bias if a large number of shots is used. Even for the ideal simulator there is a spread in the recovered angles caused by the finite sample size (shot noise). As the level of noise increases in panels (b)-(d), the position of the blue bars deviates more from the ideal red dashed line, and the spread on α^{meas} for an individual symbol increases. The distortions correction function $g(.)$, defined in Eq. (16) in the Main Text, is determined by the horizontal dotted lines, which indicate the

heuristic intervals that map the measured angle to the most probable input symbol. The edges of these heuristic intervals are chosen as the average of two consecutive blue bars. The lookup table for the distortion correction has to be precomputed through calibration, which requires additional shots, but it can then be applied to remove the bias of new measurements on **QCrank** circuits of data with similar characteristics. The dynamic range (D_r), defined in Eq. (14) in the Main Text, is naturally visible in Supplementary Fig. S1 as the vertical

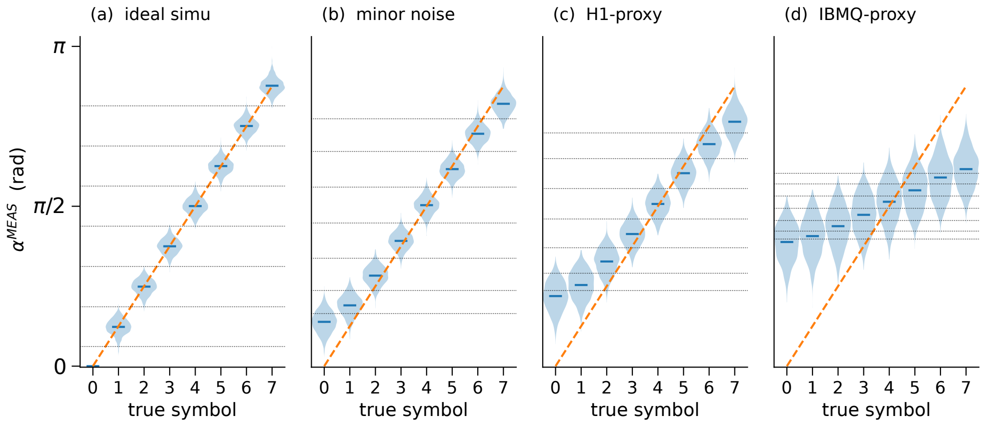

Supplementary Figure S1. Reconstruction of α^{meas} (vertical axis) for **QCrank** *experiment #5* with $3 \cdot 10^3$ shots and for four noise models listed in Supplementary Table S2 are shown on panels (a)-(d). The true values of symbols (a.k.a. discrete inputs x) are on the horizontal axis. The diagonal dashed red line marks the mathematically correct ideal reconstruction (Eq. (5) in the Main Text) and it is the same for all 4 panels. The horizontal dotted lines mark adaptive calibration thresholds used for more accurate reconstruction following Eq. (11) in the Main Text. The width of the violin plots denotes PDF α^{meas}.

distance between the first and the last blue bars scaled to the full range of π. The RVF, defined in "Methods" in the Main Text, is the area of each violin that is contained between the respective threshold lines (dotted lines) assigned to a given symbol. The final 2 rows in Supplementary Table S2 summarize D_r and RVF obtained from the data shown in Supplementary Fig. S1.

Fidelity of QCrank as a function of shots (Experiment #6) and address qubits (Experiment #7)

For *Experiment #6*, we use the same **QCrank** setup used in the previous experiment but we vary the number of shots. Supplementary Fig. S2(a) shows the measured RVF as a function of the number of shots for the four different noise models. The RVF improves with increasing number of shots, but the rate of improvement depends on the level of noise. Furthermore, with increasing noise, the RVF saturates at a lower fidelity and an RVF of 1 is only achieved for the noise-free simulator. In other words, running more shots does not compensate the higher noise and sees a diminishing improvement in RVF. In **QCrank** *Experiment #7*, we start again with the setup used in *Experiment #5*, but we maintain the number of shots constant at $3 \cdot 10^3$. Instead, we increase the number of address qubits, or equivalently, the CX depth. The measured RVF is shown in Supplementary Fig. S2(b) as a function of the CX-cycles depth of the final circuit. The RVF degrades with increasing address count due to the circuit being longer and there being fewer shots available per address. Consequently, the measured probabilities are less accurate. This effect is more pronounced in the presence of noise.

Fidelity of QBArt as a function of shots (Experiment #8)

In this experiment, we perform a similar analysis of **QBArt** with majority voting suppressing the noise. The experimental setup is as follows: we use a **QBArt** circuit with 5 address qubits and 10 data qubits that has an information capacity of $2^5 \times 10 = 320$ classical bits. We use a random sequence of 320 bits and the same 4 noise models as before (see Supplementary Table S2). Supplementary Fig. S3(a) shows the RVF as a function of the number of shots. For the noise-free, minimal noise, and H1-proxy models, the fidelity converges to 1.0 for $\mathcal{O}(10^3)$ shots or fewer. For the IBMQ-proxy noise model, the RVF remains low even if an order of magnitude more shots are used. With this noise model, the bit strings are too corrupted for the majority voting technique to work. Supplementary Fig. S3(b) shows the RSF, which measures if the *full* sequence is retrieved correctly, again for the different noise models. This experiment shows that a perfect recovery is possible using only a moderate number of shots as long as the noise-level is not too high.

Comparing Supplementary Fig. S3 with Supplementary Fig. S2 shows that **QBArt** requires significantly fewer shots to achieve a similar RVF. This is due to the sparser encoding and the data encoded in basis states, i.e. orthogonal states, compared to superpositions. Furthermore, as illustrated by the experiments in the main text, **QBArt** is more suitable for quantum data processing algorithms that act on the binary data representation, compared to the angle representation used in **QCrank**.

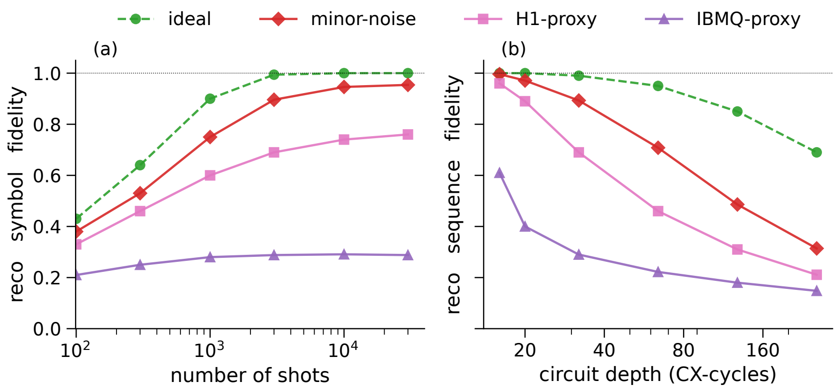

Supplementary Figure S2. Simulated **QCrank** symbol fidelity for the 4 models of noise listed in Supplementary Table S2. (a) *Experiment #6* shows fidelity dependence on the number of shots, while the problem size is constant. (b) *Experiment #7* shows fidelity as a function of the size of the **QCrank** addresses space, while the number of shots is kept constant.

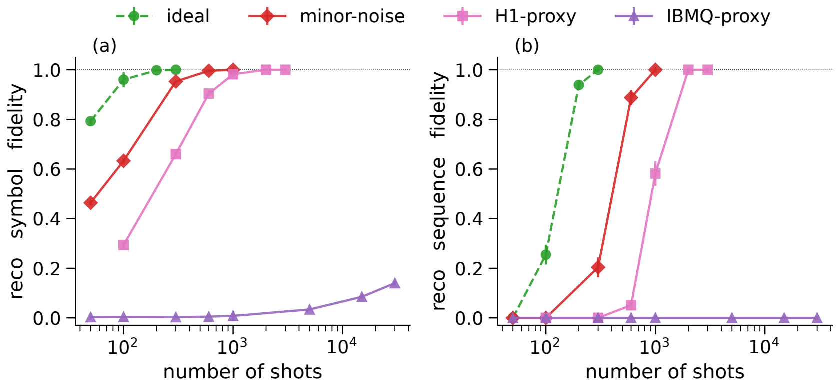

Supplementary Figure S3. Simulated **QBArt** reconstruction fidelity for **QBArt** *Experiment #8* assuming various noise magnitudes. (a) single value fidelity (b) whole sequence fidelity.

ECG waveform time-series (Experiment #9)

In this final experiment, we encode and recover a waveform consisting of 64 values in 6-bit resolution using our **QBArt** encoding. We generate a synthetic electrocardiogram (ECG) signal shown in Supplementary Fig. S4(a). This waveform is digitized into a sequence of 64 6-bit integers, shown as solid red in Supplementary Fig. S4(b), and used as the **QBArt** input. The dashed black line in the same figure shows the recovered ECG signal using majority voting. The simulation is run on the Quantinuum H1-1E emulator and correctly recovers 63 out of the 64 input values using 2000 shots. The **QBArt** circuit uses 6 address qubits and 6 data qubits and has the depth of the transpiled circuit is 64 CX gates. Our other experiments suggest that the actual Quantinuum H1-1 QPU would deliver a similar performance using 150% of the shots of the simulator.

Additional background information

Shots count requirement for QBArt

The output can be accurately recovered for **QBArt**, but with a probability that depends on the number of used shots and on the length of the sequence. The fundamental notion is the minimal number of appearances of each address sub-string (M_{min}) during multi-shot measurements. On average, each **QBArt** address is measured with the same probability. The Poisson distribution $f(x,\lambda)$ governs the number of appearances of an address. The *lower cumulative distribution*, $P(x,\lambda)$, describes the probability of detecting not more than x occurrences given the average λ:

$$P(x,\lambda) = \sum_{t=0}^{x} f(t,\lambda). \quad (5)$$

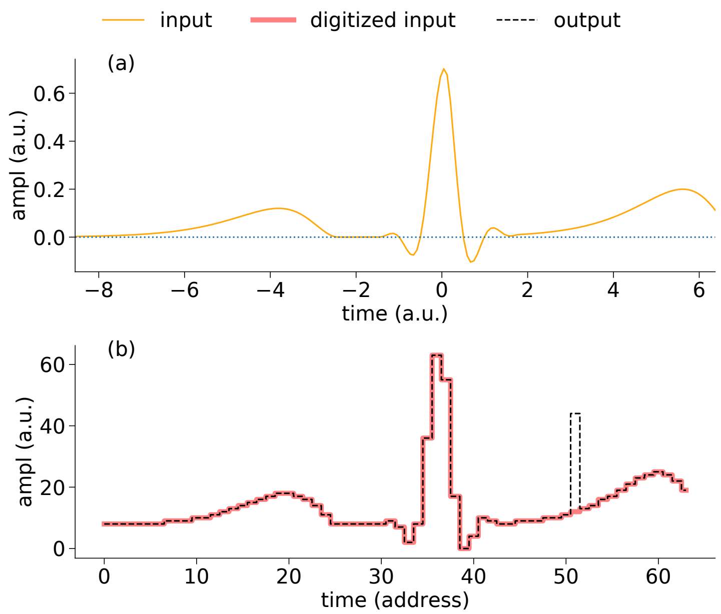

Supplementary Figure S4. Encoding of an ECG signal using **`QBArt`** on the Quantinuum H1-1E emulator. The 12-qubit **`QBArt`** circuit is executed for $2 \cdot 10^3$ shots. (a) synthetic ECG signal. (b) digitized input is shown as a solid line and the reconstructed signal is shown as a dashed line.

In our case, λ is the ratio of the number of shots per circuit to the number of **`QBArt`** addresses. For an ideal QPU, we want each address to appear at least once ($M_{min} = 1$), which will happen with probability $1 - P(0, \lambda)$. For a NISQ device, we will need $M_{min} > 1$ to allow for a sufficient number of appearances of the data-bits string at a given address, such that, more than once, the measured data bit-string is the correct one and the majority voting method selects this bit-string. It is possible to state the inverse case, i.e. the hardware agnostic problem, as follows. How many shots per address, λ, are required to achieve some value of M_{min}, while accepting some probability of failure per address, F_{addr}? Supplementary Fig. S5(a) shows analytical results of $\lambda(F_{addr}; M_{min})$ for 3 choices of M_{min}. There is a weak penalty for requiring a larger M_{min}. In the case of **`QCrank`**, we want the whole data sequence, meaning the values at all addresses, to be reconstructed correctly. At the first order, the probability of seeing less than M_{min} appearances (F_{circ}) in any of L addresses equals $L \cdot F_{addr}$. Supplementary Fig. S5(b) shows the necessary number of shots per **`QBArt`** circuit as a function of the number of addresses for selected pairs of (M_{min}, F_{circ}). The $M_{min} = 1$ results relate to an ideal QPU. It shows that we need only 350 shots per **`QBArt`** circuit with 32 addresses to obtain the correct answer with the probability of 99.9%, regardless of the number of data qubits. For a NISQ device with H1-1 QPU fidelity level, we may target $M_{min} = 8$, which requires 800 shots instead. The dependence of the total number of shots on the failure probability is rather weak.

Classical circuit for DNA matching

DNA is a sequence of *codons* consisting of three *nucleotides*. Given that 4 types of nucleotides exist, the *base 64* codon requires 6 classical bits to be encoded. Therefore, a reference classical circuit comparing two codons (Supplementary Fig. S6) must have 12 input bits, labeled $a_0, \ldots, a_5, b_0, \ldots, b_5$. The first 6 *XNOR* gates return 1 if there is a match between their two input bits. The following 5 *AND* gates aggregate this information to a single bit m_0, set to 1 if all 6 pairs of inputs match. The intermediate output of XOR is accessible via bits $p_0, \ldots, p_5$. The p_i bits can be used as input to the following Hamming weight circuit (not shown) producing the Hamming distance between the two input codons. For the binary encoding of quantum data, there is a correspondence between a classical *XOR* gate and a quantum *CX* gate. Similarly, a classical *AND* gate maps to a quantum *Toffoli* gate. We exploit this correspondence to construct the quantum circuit in Fig. 4 in the Main Text with almost identical topology as the classical one in Supplementary Fig. S6.

Classical circuit for Hamming weight

The Hamming weight of a bit-string is the number of 1s in the bit-string. For completeness, we show the classical circuit computing the Hamming weight for a 3-bit input in Supplementary Fig. S7. It can be compared with the equivalent quantum circuit. To highlight again the analogy between classical and quantum gates, the numbers inside the classical gates in Supplementary Fig. S7 enumerate the equivalent quantum gates in Fig. 5 in the Main Text. It is easy to verify that the 4 quantum gates from Fig. 5 in the Main Text implement the 3-bit Hamming weight truth table shown here.

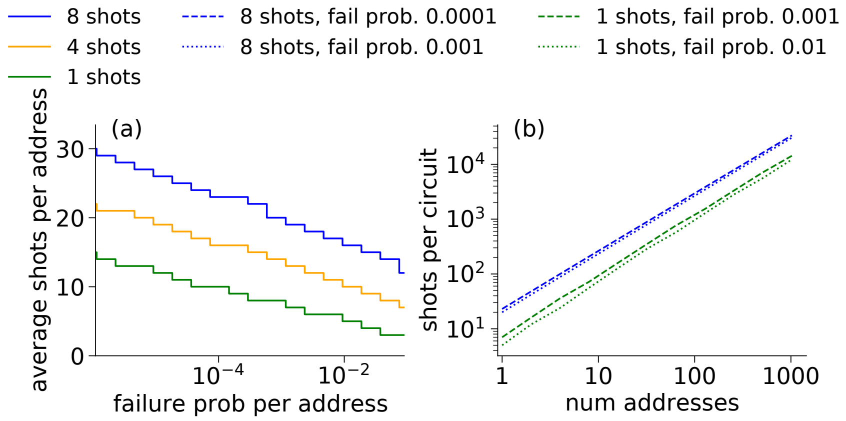

Supplementary Figure S5. Relationship between the necessary number of shots and the number of **QBArt** addresses. (a) average shots per address sufficient for the 3 choices of minimal number of shots to be guaranteed with probability above 99.9%. (b) total shots per circuit for several choices of minimal number of shots per address and confidence levels.

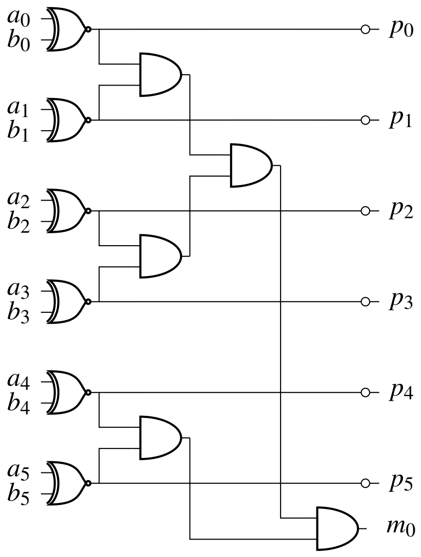

Supplementary Figure S6. Classical circuit using 6 XNOR, 5 AND, and 6 NOT gates setting bit m_0 to true if the two 6-bit input registers $a_0, \ldots, a_5$ and $b_0, \ldots, b_5$ are equal.

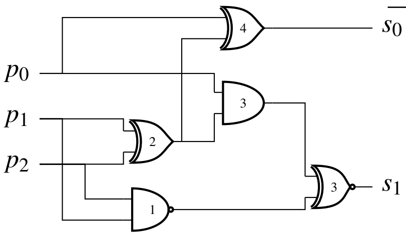

p_2	p_1	p_0	s_1	s_0
0	0	0	0	0
0	0	1	0	1
0	1	0	0	1
0	1	1	1	0
1	0	0	0	1
1	0	1	1	0
1	1	0	1	0
1	1	1	1	1

Supplementary Figure S7. (Left) Classical gates computing 3-bit Hamming weight. Logical expressions: $s_0 = p_0 \oplus p_1 \oplus p_2$, where $\oplus$ denotes *modulo 2* addition and $s_1 = \overline{p_0(p_1 \oplus p_2) \oplus \overline{p_1 p_2}}$. The numbers inside classical gates map to equivalent quantum gates in **??**. (Right) Truth table.

References

1. Möttönen, M., Vartiainen, J. J., Bergholm, V. & Salomaa, M. M. Quantum circuits for general multiqubit gates. *Phys. Rev. Lett.* **93**, DOI: 10.1103/PhysRevLett.93.130502 (2004).

2. Amankwah, M. G., Camps, D., Bethel, E. W., Van Beeumen, R. & Perciano, T. Quantum pixel representations and compression for N-dimensional images. *Sci. Rep.* **12**, 7712, DOI: 10.1038/s41598-022-11024-y (2022).

3. Nielsen, M. A. & Chuang, I. L. *Quantum Computation and Quantum Information* (Cambridge University Press, Cambridge, 2010).

4. Vidal, G. & Dawson, C. M. Universal quantum circuit for two-qubit transformations with three controlled-not gates. *Phys. Rev. A* **69**, 010301, DOI: 10.1103/PhysRevA.69.010301 (2004).
